# Shannon Entropy Analysis of a Nuclear Fuel Pin Under Deep Burnup

**DOI:** 10.3390/e26121124

**Published:** 2024-12-22

**Authors:** Wojciech R. Kubiński, Jan K. Ostrowski, Krzysztof W. Fornalski

**Affiliations:** 1 Faculty of Physics, Warsaw University of Technology, ul. Koszykowa 75, 00-662 Warszawa, Poland; wojciech.kubinski@ncbj.gov.pl (W.R.K.); jan.ostrowski2.dokt@pw.edu.pl (J.K.O.); 2 National Centre for Nuclear Research (NCBJ), ul. Andrzeja Sołtana 7/3, 05-400 Otwock-Świerk, Poland

**Keywords:** entropy, nuclear reactor, nuclear fuel, deep burnup, Monte Carlo simulations

## Abstract

This paper analyzes the behavior of the entropy of a nuclear fuel rod under deep burnup conditions, beyond standard operational ranges, reaching up to 60 years. The evolution of the neutron source distribution in a pressurized water reactor (PWR) fuel pin was analyzed using the Monte Carlo method and Shannon information entropy. To maintain proper statistics, a novel scaling method was developed, adjusting the neutron population based on the fission rate. By integrating reactor physics with information theory, this work aimed at the deeper understanding of nuclear fuel behavior under extreme burnup conditions. The results show a “U-shaped” entropy evolution: an initial decrease due to self-organization, followed by stabilization and eventual increase due to degradation. A minimum entropy state is reached after approximately 45 years of pin operation, showing a steady-state condition with no entropy change. This point may indicate a physical limit for fuel utilization. Beyond this point, entropy rises, reflecting system degradation and lower energy efficiency. The results show that entropy analysis can provide valuable insights into fuel behavior and operational limits. The proposed scaling method may also serve to control a Monte Carlo simulation, especially for the analysis of long-life reactors.

## 1. Introduction

### 1.1. Nuclear Power

Nuclear power is one of the most important solutions for low-carbon electricity production and mitigation of climate change. Several hundred nuclear reactors generate energy for hundreds of millions of human beings worldwide. More than that, dozens of new nuclear projects are currently underway around the world. This creates a valuable place for many scientists who are working for more effective and safer nuclear power in the future.

The nuclear reactor is a physical, complex system, far from thermodynamical equilibrium, where the neutron flux maintains its operation. In a relatively short period of time, the nuclear reactor behavior can be treated as a non-equilibrium steady state, where all thermodynamical parameters are generally constant [1]. However, due to the long-term nuclear fuel burnup process, the permanent global steady state cannot be typically reached, which can be verified by computational simulations and entropic analyses.

### 1.2. Shannon Entropy in Monte Carlo Simulations

One of the methods for solving neutron transport problems, alongside deterministic ones, is the probabilistic method, based on Monte Carlo simulations. In this approach, instead of directly solving transport equations, a simulation of the history of many particles is carried out. Then, through the appropriate random sampling of possible reactions, based on available probability distributions, the statistics and properties of the system are determined [2].

In Monte Carlo neutron transport calculations, neutron production sources are sampled based on a fission point distribution. In cases where the distribution of fission sources in the simulated material is not known a priori, an initial guess is made, typically assuming a uniform distribution [3]. During the initial cycles of the simulation (referred to as inactive cycles), the simulation runs without contributing to the final results, allowing time for the source distribution to converge to a realistic state. This ensures that the results are not biased by the initial guess of the source distribution. Therefore, a selected number of neutron histories per cycle (typically 10,000 to 100,000, depending on the system size [4]) is simulated to observe where neutrons ultimately emerge and from where histories should be initiated in the main calculations. After reaching statistical convergence (simulating the appropriate number of inactive cycles), the distribution satisfies the equation [3,5]

(1) $$Hs\left(\vec{r},E\right)=ks\left(\vec{r},E\right)$$

where *k* is the eigenvalue (with its highest value related to the effective neutron multiplication factor $k_{eff}$), $s(\vec{r},E)$ is the eigenfunction describing the concentration of fission neutrons (with energy *E* at position $\vec{r}$), and *H* is the fission operator [5]:

(2) $$HS\left(\vec{r},E\right)=\int_{0}^{\infty }\int_{V}f\left(\vec{r^{\prime }}\to \vec{r},E^{\prime }\to E\right)\phi \left(\vec{r^{\prime }},E^{\prime }\right)d^{3}r^{\prime }dE^{\prime }.$$

In this equation, $\phi (\vec{r^{\prime }},E^{\prime })$ is the neutron flux, and the function $f(\vec{r^{\prime }}\to \vec{r},E^{\prime }\to E)$ is used to describe the expected number of fission neutrons produced at position $\vec{r}$, of the energy *E*, resulting from a fission neutron born at $\vec{r^{\prime }}$ with energy $E^{\prime }$, assuming isotropic emission of the fission neutrons [5]. In general, in Monte Carlo simulations, the convergence of the eigenvalue $k_{eff}$ is monitored as the main criterion for calculation convergence. However, this may prove insufficient, as in some systems it may happen that the convergence of the neutron multiplication factor appears before the source convergence [6]. Therefore, entropy, in the context of information entropy (Shannon entropy), is often used in Monte Carlo simulations (f.i. in codes such as Serpent2 and MCNP [6]) as an additional criterion of the convergence of the neutron sources in fissile material. Shannon entropy is given by the equation

(3) $$S=-\sum_{i}p_{i}\cdot log\left(p_{i}\right),$$

where $p_{i}$ is the probability of finding a neutron source in cell *i*, which is an element of a rectangular grid $n_{x}\times n_{y}\times n_{z}$, spread throughout the system. Shannon entropy has wide applications in describing all processes that may involve randomness. It is used in problems of information theory, as well as in fields such as data compression and analysis [7,8], cryptography [9], physics, biology, and economics [10], machine learning [11], and as a convergence criterion for optimization algorithms [12,13,14].

When a Monte Carlo simulation without a predefined source distribution starts with a random, uniform sampling, the source is characterized by a high level of disorder and a high value of entropy. As the cycles progress and the source converges, the system, becomes, in a sense, more ordered, and its entropy decreases to a certain value. It is therefore important to perform an adequate number of inactive cycles with sufficient neutrons to ensure the source distribution’s convergence, as described previously. On the other hand, these simulations do not contribute to the statistics of the final calculations, so this number should not be too large. It should be emphasized that when analyzing the convergence of the Shannon entropy, it is necessary to use an appropriate number of neutrons simulated per cycle. This means that to ensure adequate statistics, the number of particles must be significantly greater than the number of nodes, i.e., in general for a 3D simulation,

(4) $$N\gg n_{x}\cdot n_{y}\cdot n_{z}.$$

If this condition is not met, the entropy rapidly converges to a certain value, dependent only on the number of particles *N* and the grid size, showing an illusory convergence of the sources.

### 1.3. Nuclear Fuel Burnup

As the reactor cycle progresses, the nuclear fuel burns up, and its isotopic composition changes. The nuclei undergo nuclear reactions, absorb neutrons, split, decay, and transmute into others. The released energy is used to express the fuel burnup, defined as MWd/kgU (megawatt days of released energy per kilogram of uranium) or EFPDs (effective full power days). The evolution of nuclide concentrations during burnup is described by the Bateman equations [2]:

(5) $$\begin{array}{cc} \frac{dN_{X,i}\left(t\right)}{dt}= & -\lambda_{X}N_{X,i}\left(t\right)-\int_{0}^{\infty }\sigma_{aX}\left(E\right)N_{X,i}\left(t\right)\phi_{i}\left(E,t\right)dE\\ & +\sum_{Y}N_{Y,i}\left(t\right)[\lambda_{Y}+\int_{0}^{\infty }\sigma_{cY}\left(E\right)\phi_{i}\left(E,t\right),dE\\ & +\gamma_{Y/X}\int_{0}^{\infty }\sigma_{fY}\left(E\right)\phi_{i}\left(E,t\right)dE\\ & +\beta_{Y/X}\int_{0}^{\infty }n_{Y}\left(E\right)\sigma_{fY}\left(E\right)\phi_{i}\left(E,t\right)\;dE] \end{array}$$

where $N_{X,i}$ is the concentration of nuclide *X* in region *i*, that decreases due to the following:

- $\lambda_{X}N_{X,i}\left(t\right)$: radioactive decay of nuclide *X*;
- $\sigma_{aX}\left(E\right)N_{X,i}\left(t\right)\phi_{i}\left(E,t\right)$: absorption of a neutron.

And increases due to the following:

- $\lambda_{Y}$: decay of nuclide *Y* into nuclide *X*;
- $\sigma_{cY}\left(E\right)\phi_{i}\left(E,t\right)$: neutron capture of nuclide *Y* that leads to creation of nuclide *X*;
- $\gamma_{Y/X}\sigma_{fY}\left(E\right)\phi_{i}\left(E,t\right)$: fission of nuclide *Y* that leads to creation of nuclide *X*;
- $\beta_{Y/X}n_{Y}\left(E\right)\sigma_{fY}\left(E\right)\phi_{i}\left(E,t\right)$: created as a precursor of a delayed neutron (due to fission of nuclide *Y*).

Along with changes in nuclide concentrations, the entire system is modified, including its $k_{eff}$ value, neutron flux distribution, and neutron source locations, which simultaneously affect the value of the Shannon entropy.

## 2. Materials and Methods

### 2.1. Entropy Analysis

As described in Section 1.3, during nuclear fuel burnup, the concentration of nuclides changes according to Equation (5). The system, therefore, modifies itself, and the distribution of new neutron sources evolves, which in particular can cause a change in the value to which Shannon entropy (defined as Equation (3)) converges. This study aims to investigate how the distribution of neutron sources behaves during the burnup of a fuel pin of a typical power PWR (pressurized water reactor) reactor. In the pin, on the one hand, we start with a homogeneous fuel distribution, so the entropy of the sources should be at its maximum at the beginning of the cycle. On the other hand, effects such as self-shielding and neutron thermalization may cause the initial distribution of sources to differ significantly from the distribution of fissile material. The neutron source distribution, therefore, cannot be easily guessed upfront. To analyze the problem, the typical burnup range of a pellet (<100 MWd/kgU, or below 7 years) was simulated, with extended analysis with burnup up to 800 MWd/kgU (around 60 years), which exceeds the applicability limits of the fuel material. This was performed to explore aspects of statistical physics and to gain a deeper understanding of the process. In the analysis, 100 cycles of $10^{5}$ neutrons were simulated for each burnup step, each time checking the value to which the entropy converged.

### 2.2. Numerical Model

**Figure 1:**
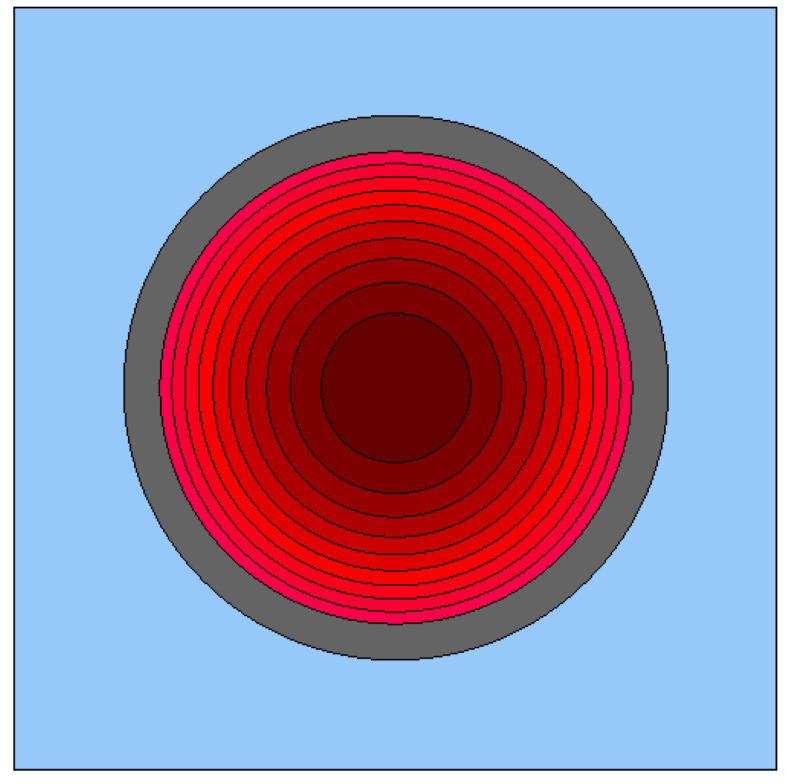
The scheme of the fuel pin’s horizontal cross-section geometry. The red region is the fuel pellet divided into concentric rings of equal cross-sectional areas, and the gray region is the cladding.

In the study, the simplest case was analyzed, namely, the two-dimensional burnup of a typical fuel rod from a PWR, taken from the example in [15]. The pin consisted of a fuel pellet of 3% enriched uranium dioxide with a radius of 0.412 cm, covered with approximately 0.63 mm of Zircaloy cladding (see Table 1). The power density was taken from the example and was set to 40 kW/kgU. To simplify the model, it was assumed that there was no gap between the fuel and the cladding. The pin was surrounded by water. A single fuel pin cell was simulated with periodic boundary conditions (see Figure 1).

For computational reasons (solving the Bateman Equation (5) separately for each region), the pin was divided into K=10 concentric rings (red regions in Figure 1), with equal surface areas (a typical way in this procedure). Then, the radii of each of them satisfied the relationship $r\left(k\right)=r_{0}\sqrt{k/K}$, where $r_{0}=0.412$ cm is the outer radius of the pellet.

### 2.3. Shannon Entropy Analysis

For fresh nuclear fuel, the radial burnup profile within a single pin is initially uniform, reflecting an even distribution of fissile material. Over time, the radial burnup evolves due to variations in neutron interactions and the accumulation of neutron-absorbing fission products (“poisons”). This results in changes to the distribution of neutron sources and a corresponding impact on the Shannon entropy. The entropy is directly related to burnup (BU), as changes in fuel composition directly influence the location of neutron sources. This relationship is time-dependent, with *S*(BU)∼*S*(*t*), linking burnup and entropy evolution over the reactor’s operational period. To analyze entropy changes, a non-equilibrium approach was adopted, where the rate of entropy change (ΔS or dS) was calculated in the context of Shannon entropy as a function of burnup, which is proportional to time. This study aims to analyze changes in the entropy of the neutron source distribution in the context of burnup, particularly for very high burnup values and significant, long-term changes in nuclide concentrations and fuel parameters.

Two methods were employed for the analysis:

1. **Standard serial simulation:** This approach used the conventional Serpent2 code to simulate successive generations of $N=10^{5}$ neutrons. Each generation builds on the results of the previous one, reflecting the natural progression of burnup.
2. **Proportional neutron scaling**: This novel method involved adjusting the neutron source count between generations inversely proportional to the fission rate, ensuring consistent neutron source statistics across all time points. This adjustment was designed to normalize statistical variations and improve the robustness of entropy calculations over the simulation period.

Both methods were tested and are discussed in more detail in the following subsections.

## 3. Results

### 3.1. Burnup Calculations

The burnup calculations were performed for the described two-dimensional pin model, covering 23 burnup steps, with $10^{7}$ neutrons per step. Due to the complexity of Monte Carlo burnup calculations, ensuring adequate statistical accuracy is crucial.

The burnup steps covered two ranges: the first, a standard operational range for the pin, from 0 to 100 MWd/kgU (7 years of full power operation); and the second, an extended burnup range up to 800 MWd/kgU (60 years), reaching a very low value of the neutron multiplication factor ($k_{eff}\approx 0.4$) and the computational capabilities of Serpent2 (above this burnup value, the fissile material content did not allow the simulation to continue). This extended range goes far beyond the practical applicability of fuel material due to numerous technical limitations, such as mechanical damage and embrittlement [16]. However, as noted, this range was explored for the purpose of statistical physics analysis.

For each burnup step, the concentrations of nuclides were determined. Then, for each state, static calculations were performed to determine the distribution of the fission rate density, the distribution of the thermal neutron flux, and the convergence of the Shannon entropy.

**Figure 2:**
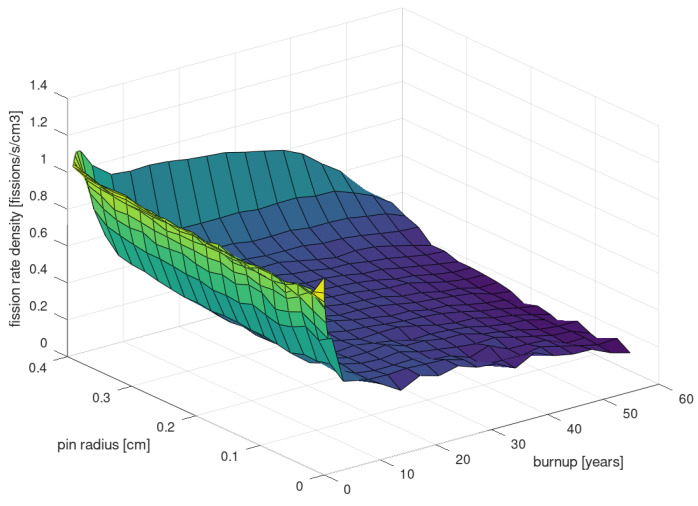
A 3D plot of fission rate density as a function of nuclear fuel pin diameter (in cm) and fuel burnup (expressed in years).

**Figure 3:**
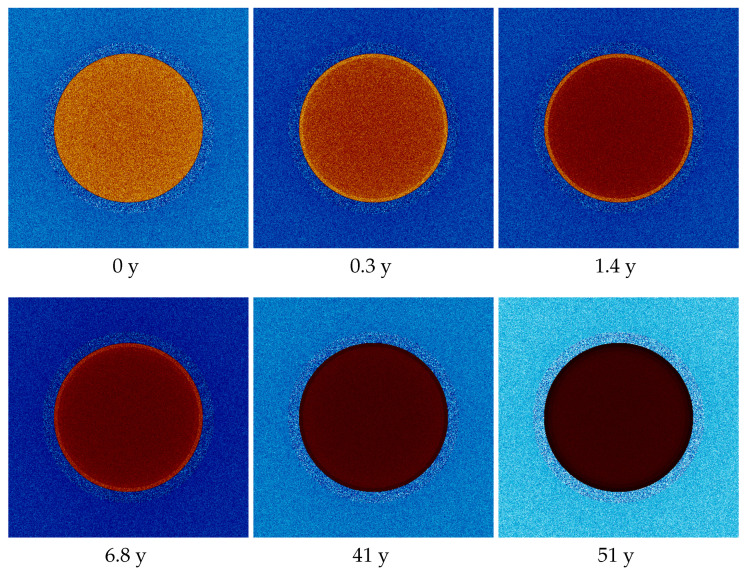
Fission source distributions (red and yellow) and thermal neutron distributions (blue and white) during the depletion. Graphs in the first line present the typical burnup range, and graphs in the second line present a highly extended one.

Figure 2 presents the radial distribution of the fission rate density (number of fissions per second per cm^3^), depending on the burnup value. In general, the fission rate of a region *V* can be defined as

(6) $$\dot{F}\left(V\right)=\int_{0}^{\infty }\int_{V}\Sigma_{f}\left(\vec{r},E\right)\phi \left(\vec{r},E\right)dr^{3}dE$$

where $\phi (\vec{r},E)$ is the neutron flux at position $\vec{r}$ and energy *E*, and $\Sigma_{f}\left(\vec{r},E\right)$ is the macroscopic cross-section, related to the probability of fission. The fission rate density of a fresh fuel pin begins with a relatively uniform distribution, but after just a few days (>0.1 MWd/kg), the fuel starts to burn more intensively at the edges of the pellet (more fission at the edge). This effect is associated with the accumulation of poisons inside the pin, and the easier access of thermalized neutrons to the edges of the pin. Then, as seen in Figure 2, the fission rate quickly increases at the edge of the pin and decreases in the center. However, after around 1.5 years (>20 MWd/kgU), fissile material starts to become deficient at the edge of the pin, and the situation begins to reverse. The fission rate at the edge starts to decrease in favor of an increase in the central part of the pin, and the distribution begins to take on a completely opposite shape (see the visualization of thermal neutron flux and sources for selected burnups presented in Figure 3).

**Figure 4:**
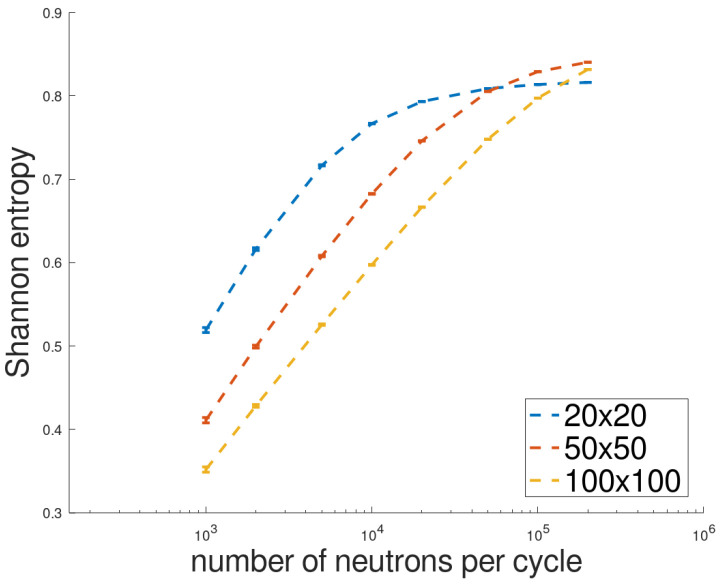
Convergence of Shannon entropy for different numbers of neutrons per cycle. Simulations of 100 cycles were calculated for fuel initial state using three different grids: 20 × 20, 50 × 50, and 100 × 100.

Before conducting the full analyses, the dependence of the value to which the Shannon entropy converges on the mesh density on which it is calculated and the number of neutrons per cycle was checked in order to select the appropriate values. For the sake of simplicity, the analyses were conducted for fresh fuel and three selected grids: 20 × 20, 50 × 50, and 100 × 100. Figure 4 presents the results; they show that the value of entropy strongly depends on the grid used and the number of simulated neutron histories (and thus the number of sources). A small grid leads to faster convergence but to a lower value. A larger grid increases the value of entropy but slows down convergence. Additionally, the graph indicates that entropy converges to a certain value as the number of neutrons increases. In this study, a compromise between convergence and the value of entropy was made, and a 50 × 50 grid was chosen, which seemed to be sufficient for a small system such as a fuel pin.

### 3.2. Constant Size of Initial Neutron Population

**Figure 5:**
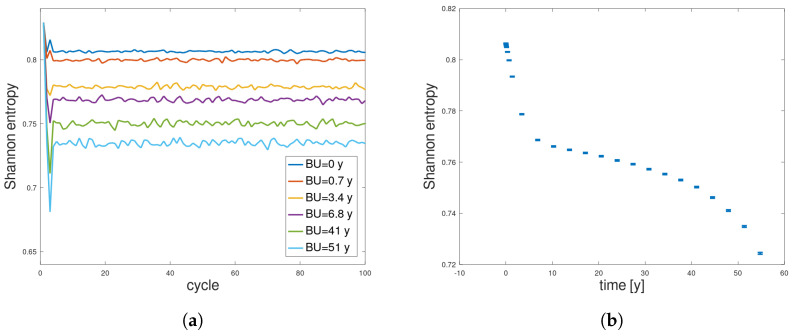
Constant neutron population: Shannon entropy convergence in terms of (**a**) simulated cycles, presented for selected burnup values, and (**b**) converged value of Shannon entropy, with one standard deviation, presented as a function of burnup.

In this section, an analysis of the convergence of the Shannon entropy was performed for each determined state during burnup (for each burnup step), for 100 cycles (+10 inactive cycles), and $10^{5}$ neutrons each. Figure 5a shows the convergence of the Shannon entropy during the simulated cycles at selected burnups. The graph indicates that entropy stabilizes within just a few initial cycles and oscillates around a certain average value. Additionally, from Figure 5a,b it can be seen that the greater the burnup, the lower the final average value of the Shannon entropy. This could suggest that the system is organizing itself, and from a more homogeneous source of neutrons, a more refined structure begins to form, resulting in a decrease in the Shannon entropy, which is partly confirmed in Figure 3. However, analyzing Figure 2, we can observe another effect—along with the burnup, effectively each of the simulated neutron histories leads to fission less frequently (the effective neutron multiplication factor from Equation (1) falls below 1), which results in a smaller number of available sources and a decrease in fission rate density. The smaller number of sources leads to reduced statistics, and this (according to Figure 4) may lead to an artificial decrease in the value of the entropy.

While the number of initiating particles is constant for each time step of the simulation, the number of neutron sources used for the Shannon entropy calculation is not, due to a decreasing fission rate. With a very limited number of neutron sources inside the pin, a number comparable to the number of cells in which the neutrons are counted, the macroscopic measurement of entropy is distorted significantly at this level by stochastic events. In other words, the chance for a few neutrons to equally split between a large number of cells is relatively small, which is impossible if there are fewer neutrons than cells to be fitted. Therefore, it is important to verify the findings of this analysis with the possibility of accumulation of results of multiple simulations before calculating the entropy. It would be expected that at that point the second slope should change to a decrease in entropy and possibly reach its maximum. To avoid that problem, we simply proposed to keep the neutron sources at a constant level.

### 3.3. Scaled Size of Initial Neutron Population

The analysis performed in the previous section has a fundamental bias due to the decreasing number of sources included in the final cycles. The statistically significant decrease in neutrons can affect entropy. To avoid this problem, it was decided to scale the total simulated number of neutron histories per cycle and provide constant statistics during the entropy calculation. To ensure that the number of sources used to calculate the distribution entropy remains constant, the population size in the *i*-th burnup step $\tau_{i}$ was scaled with fission rate (Equation (6), an equivalent of the distribution in Figure 2, integrated over the radius):

(7) $$N\left(\tau_{i}\right)=N_{0}\cdot \frac{\dot{F}\left(\tau_{0}\right)}{\dot{F}\left(\tau_{i}\right)}$$

where $N_{0}=10^{5}$ and $\dot{F}\left(\tau_{0}\right)$ are the initial size of the population and initial fission rate. In this way, the simulated population of neutrons was gradually increased so that the final number of obtained sources remained constant.

**Figure 6:**
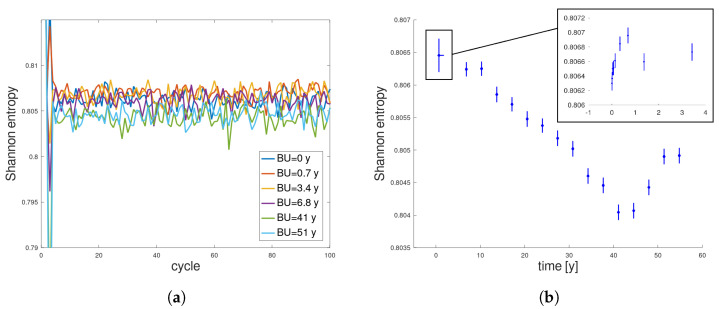
Scaled neutron population: Shannon entropy convergence in terms of (**a**) simulated cycles, presented for selected burnup values, and (**b**) converged value of Shannon entropy, with one standard deviation, presented as a function of burnup (with averaging of values for the first 8 burnup steps).

**Figure 7:**
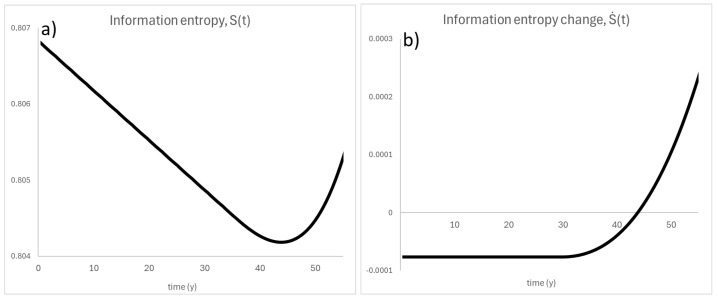
(**a**) The approximated function of information entropy related to time; see Figure 6b. Please note that the function S(t) created the “U-shape”, similarly to every far-from-equilibrium complex system. (**b**) The first derivative of information entropy, namely, the entropy change over time, which changes the sign at around 45 years of operation.

Figure 6a,b present updated Shannon entropy plots, respectively, as a function of cycles and a function of burnup, for the same burnup steps as before. First of all, the fluctuations are observed during the first 4 years of operations (so for a typical period of nuclear fuel operation in PWR or BWR reactors). Such fluctuations of entropy are possible for regions of $k_{eff}>1$ where the number of sources exceeds the number of simulated particles, so the initial locations of the neutrons in the next population are drawn randomly from the positions bank. For this reason, it was decided to average entropy values in that first period of 4 years (both in entropy and time space, presented with one asymmetrical standard deviation in the small graph in Figure 6b). After that, the information entropy decreases. This decrease, however, is quasi-linear, which is typical for regularly multiplying systems [17,18,19], and a nuclear fuel can be considered as such a self-multiplying system. After 45 years of operation, the entropy value reaches its extreme minimal value and the entropy change is zero (see Figure 7b). Then, the entropy value increases. The reason for this behavior is that the fuel burnup system absorbs nuclear binding energy and transforms it into neutrons and other products like heat. While the energy source is depleting and the obscure outside interference is decreasing, the fuel pin is closer to total burnup—the lowest entropy cannot be sustained. The fuel pin has dissipated most of it energy and the neutron interactions are closer to those of a perfect gas. While comparing the results presented in Figure 5 and Figure 6, it can be concluded that insufficient statistics was most likely to blame for the second decreasing slope in the first results.

From Figure 6a, we see that the entropy curves overlap, and their fluctuations obscure potential convergence. However, when we analyze the values to which the entropy converges, shown in Figure 6b, we see a trend that is entirely different from what was previously observed (Figure 5b). Entropy changes are now significantly smaller, though still beyond statistical uncertainty. This time, we see that the statistics are stable because the standard deviation remains constant at each step. The entropy initially strongly oscillates (see the small plot in Figure 6b), then begins to decrease in a largely linear fashion, reaches a minimum, and increases at the end. Let us call this shape a “U-shape”, which will be commented on later. This aligns with predictions and charts depicting source distributions for different burnup values (Figure 3). The sources start with a uniform distribution of high entropy, then, as the fuel burns more on the pin’s edge, the sources become more ordered, causing entropy to decrease. Finally, as fissile material becomes depleted at the edge, the sources begin to concentrate in the pin’s interior, and entropy rises.

The non-trivial behavior of entropy at the very beginning of the simulation (Figure 6b) appears interesting. It seems that the value to which it converges oscillates initially, exceeding statistical uncertainty. One could even identify an increase in entropy (see the small plot in Figure 6b), which seems to be unphysical, as an initially uniform distribution should theoretically have maximum entropy. However, at the cycle’s beginning, there are many nonlinear changes in the system parameters and nuclide concentrations. For example, in the first steps of the simulation, the average number of neutrons produced per fission ($\bar{\nu }$) rises greatly. This number influences the weight assigned to each source in the source bank, thus modifying entropy. Therefore, despite a spatially uniform distribution of sources, it is possible that the weight distribution might be non-uniform (e.g., due to neutron thermalization at the pin’s edge). Finally, it is essential to remember that the concentrations used to calculate the Shannon entropy are based on earlier Monte Carlo burnup calculations, which also contain uncertainties, not included in this analysis. However, we observe that beyond the initial simulation phase, where numerous changes occur and the system stabilizes, scaling by the fission rate ensures stable statistics and enables analysis of the source distribution entropy behavior, unaffected by the decreasing number of emerging sources.

Let us go back to the specific “U-shape” presented in Figure 6b. This type of entropy behavior seems to be natural for most far-from-equilibrium thermodynamic systems: the entropy initially decreases, dS<0 (the system is self-organizing), reaches some minimum (global steady state), and eventually the entropy goes up, dS>0 (the system is going to equilibrium and simply disintegrates). Indeed, every non-equilibrium thermodynamical system is characterized by a smaller entropy value, which is lower than the surroundings or simply the equilibrium state [1,20,21,22]. This effect is only visible for simulations with a scaled population, as the statistics decrease does not mask it. As presented, for higher values of fuel burnup, the level of order increases and the entropy quasi-linearly decreases until it reaches a minimum value. This finding is consistent with other entropy analyses of multiplying systems [17,18,19], where such a temporal linearity of entropy change was obtained (dS=const<0). This is discussed in the Section 4. After a very long time, when the system reaches the global steady state, the entropy reaches its absolute minimal value (dS→0). This seems to be interpreted as showing that the nuclear reactor is operating on its minimum energetic level due to the extreme fuel burnup. This is fully consistent with Prigogine’s extension of the second law of thermodynamics [1,21]. However, due to degradation processes, the system entropy finally increases (dS>0), which is observed in Figure 6b, after approximately 45 years of nuclear reactor operation.

The entropy change is related to the free energy of the system. Nuclear fissions and the energy they provide give the possibility of self-organization of the system with entropy production and, as a consequence, the system entropy decreases. This can be abductive reasoning, indicating that the information entropy change indicates changes in the physical properties of the system. In particular, the transition of the system with the change in the sign of its entropy rate may indicate key changes related to fission and the chain reaction.

## 4. Discussion

### 4.1. The Meaning of the Entropy of Neutron Sources

In systems at equilibrium, the entropy always takes on a maximum value. In a situation far from thermodynamic equilibrium, entropy can be defined in different ways, we therefore speak of both thermodynamic (heat-related) and stochastic (probability-related) entropy. From a purely physical point of view, the two approaches are essentially equivalent and do not contradict each other. The latter is very often equated with the entropy of information and its special case Shannon entropy. In particular, in the presented paper, it should be noted that phenomena are considered in the context of the clustering (flow) of neutron sources, and not parameterized by energy distribution.

The presented paper postulates that entropy, particularly Shannon entropy, is a useful macroscopic parameter for studying advanced physical systems with energy production. In particular, the entropy of the distribution of neutrons (and their sources) in a fuel pin located in the core of a nuclear reactor is calculated. In view of this, the entropy we use is not the full thermodynamic entropy of the system, directly related to heat transfer and associated with the distribution of energy, but the information entropy describing such a physical system, which is de facto its parameter (state function). Please note that such information about the system also gives us indirect knowledge of the heat-transfer capabilities; however, a number of other parameters are also needed for their detailed calculation. The Shannon entropy alone is not sufficient for this.

Additionally, the Serpent2 code utilized in this study is based on the Monte Carlo method, which in nuclear engineering does not independently provide thermodynamic information but is usually used for neutron calculations, such as the distribution of neutron flux density or those related to the chain reaction and neutron multiplication. Thermo-hydraulic properties typically require the use of additional code; however, this study aimed to investigate what additional information Shannon entropy might carry in a broader context.

### 4.2. The Entropy Change

One has to note that the nuclear fission reaction, namely, the chain reaction, is a self-organizing and multiplying process. This makes the reactor core structure a far-from-equilibrium thermodynamical system, in which the entropy can decrease [23,24,25]. Especially, regularly multiplying systems exhibit a linear decrease in entropy, at least for some period of their evolution. This was previously observed for viruses [17,18,19]. The conducted simulations show that the nuclear reactor may behave in a similar matter. The entropy of neutron sources reaches some minimum value which can be connected with the Prigoginian minimum thermodynamic entropy production. Indeed, for extremely high fuel burnup, the chain reaction frequency is very small, as represented by Figure 6b for approximately 45 years of operation. However, after the system reaches its entropy minimum, the information entropy starts to increase. This seems to be quite natural; due to system degradation and equilibration, the entropy change becomes positive [26].

The data from Figure 6b were approximated by a polynomial line of best fit (to observe its general trend); see Figure 7a. The “U-shape” is now easier to observe. Observing the derivative of the entropy (Figure 7a), one can note the negative entropy change below 45 years of operation (Figure 7b), and positive entropy change above that. This is a simple response of the system to changing internal conditions of fuel burnup. This observation may suggest a potential physical limit to the use of nuclear fuel. The fuel is used even when the effective neutron multiplication factor drops below 1.0, which means that a given portion of the fuel is no longer capable of sustaining the chain reaction on its own; however, it can still produce energy if a neutron appears and fission occurs. A change in the sign of entropy could suggest that, despite the presence of fissile material in the fuel pin ($k_{eff}>0$), on average, the pin is unable to produce more energy than that which was supplied to it. This would be an interesting observation, suggesting that for the analyzed system, the maximum (physical) burnup limit would be around 45 years of operation at full power. Of course, technical limits and the actual use of fuel are well below this period, but intensive work on long-life reactors, especially small modular reactors [27], may shift these limits towards higher values in the future. However, it should be noted that in this study, Shannon’s information entropy was considered and applied to the distribution of neutron sources. Nevertheless, the change in the sign of entropy change is related to physical processes and definitely suggests a closer examination of this specific moment in time during the burnup process.

### 4.3. Neutron Population Scaling

The proposed method of fission rate scaling of the neutron population allows us to obtain a sufficient number of neutron sources in the late stages of the simulation. This may be crucial when collecting statistics related to parameters based on the occurrence of nuclear reactions. The Shannon entropy can be treated as an indicator of the complexity level of the studied system and may suggest the number of particles needed to sample the current state of the system. We can imagine that analyzing a system with a uniform source distribution is likely less numerically demanding than a system with a complex distribution. In such a case, unnecessary simulations of simple-to-analyze states could be avoided, allowing computational power to focus on more problematic ones.

Scaling based on the value of the fission rate seems like a reasonable choice, despite entropy fluctuations in the initial burnup phase. On the other hand, directly controlling entropy and maintaining it at a constant level could potentially be a good method for ensuring adequate statistics and for real-time control of simulated particles. This would represent a type of optimization for Monte Carlo simulations. Similar optimizations are employed by increasing the population depending on the error of the neutron source convergence [28] or selecting optimal burnup steps [29].

## 5. Summary and Conclusions

In this paper, 2D burnup calculations of a typical fuel rod were conducted, covering both a standard burnup range and a range significantly exceeding commonly used levels of fissile material, up to the computational limits of the Serpent2 code (approximately 800 MWd/kgU). Subsequently, for the calculated burnup steps, the convergence of Shannon entropy for neutron sources was investigated in two scenarios: the first simulating a constant number of neutrons per cycle; and the second applying scaling based on the fission rate, which stabilized the number of sources and maintained consistent statistics during entropy analysis.

Population scaling proved necessary, as the decrease in the number of sources led to significantly reduced statistics and an artificial drop in entropy, obscuring real effects. After stabilizing the statistics, the fuel pin, treated as a multiplying system far from equilibrium, exhibited properties typical of such statistical systems, showing similarities to the behavior of cancer cells and viruses (specifically, entropy decline, stabilization, and subsequent growth).

The identified point of zero entropy production may suggest a potential physical limit for nuclear fuel utilization and represents an intriguing subject for further analysis, particularly in the context of long-life nuclear reactors, which are currently under intensive investigation. Additionally, the analyses revealed that scaling neutron populations based on the fission rate and Shannon entropy could serve as an effective method for moderating the number of simulated neutrons and optimizing Monte Carlo computation time, which will be further explored in future research.

## Figures and Tables

**Table 1:** Composition of the fuel pin.

Material	Composition	Density [atoms/cm^3^]
Fuel pellet	U-234	$6.15\times 10^{18}$
	U-235	$6.89\times 10^{20}$
	U-236	$3.16\times 10^{18}$
	U-238	$2.17\times 10^{22}$
	C-12	$9.13\times 10^{18}$
	N-14	$1.04\times 10^{19}$
	O-16	$4.48\times 10^{22}$
Cladding	Zr (natural)	$4.24\times 10^{22}$
	Sn (natural)	$5.29\times 10^{20}$
	Fe (natural)	$3.54\times 10^{20}$

## Data Availability

All data were generated during the study.

## Abbreviations

The following abbreviations are used in this manuscript:

S	Shannon entropy
BU	Burnup (in MWd/kgU)
$k_{eff}$	Effective neutron multiplication factor
PWR	Pressurized water reactor
BWR	Boiling water reactor
MWd/kgU	Megawatt-days per kilogram of uranium
EFPDs	Effective full power days (here expressed in years)
